# Epithelial–mesenchymal transition (EMT) in vulvar cancer with and without inguinal lymph node involvement

**DOI:** 10.1007/s00432-021-03715-2

**Published:** 2021-09-08

**Authors:** Christine E. Brambs, Lars-Christian Horn, Meinhard Mende, Michael Höckel, Christine Eckey, Gesine Grit Ruth Hiller, Anne Kathrin Höhn

**Affiliations:** 1 Department of Obstetrics and Gynecology, Luzerner Kantonsspital, Lucerne, Switzerland; 2 Institute of Pathology, Division of Breast Gynecologic and Perinatal Pathology, University Hospital of Leipzig, Liebigstrasse 26, 04103 Leipzig, Germany; 3 Clinical Trail Centre and Institute of Medical Information, Statistics and Epidemiology, University of Leipzig, Leipzig, Germany; 4 Division of Gynecologic Surgical Oncology, Department of Obstetrics and Gynecology (Institute of Trier), University Hospital of Leipzig, Leipzig, Germany

**Keywords:** Vulvar cancer, EMT, p53, p16, Epithelial, Mesenchymal transition, Front of invasion, Cyclin D1, Vimentin, e-cadherin, Mesenchymal-epithelial transition, MET

## Abstract

**Purpose:**

Epithelial-mesenchymal transition (EMT) is associated with increased metastatic spread and poor prognosis. Data on vulvar carcinoma are limited.

**Methods:**

Thirty-two cases of squamous cell carcinoma of the vulva (16 with and 16 without inguinal lymph node metastases) and their lymph node deposits were evaluated for immunohistochemical expression of EMT markers (vimentin, cyclin D1, e-cadherin), p16, p53 and Ki-67. Results of EMT-immunostainings were compared to lymph node involvement and expression of p53 and p16. The micro-anatomical staining pattern for EMT markers comparing the tumor center with the front of invasion was analysed in each tumor.

**Results:**

There was no difference in the expression of EMT markers between node negative and node positive tumors. Staining for vimentin and cyclin D1 was seen within tumor cells at the front of invasion in 100 and 84.4% of the tumors, respectively. The majority of cases (68.7%) showed negative or reduced staining for e-cadherin in this micro-anatomical localization. Tumor cells within the lymph node metastases showed positive staining for e-cadherin in 75% and for cyclin D1 in 49% of the cells but were negative for vimentin in 13 out of 16 cases (81.3%). Tumors with aberrant p53 staining represented a non-significant higher vimentin but significantly higher cyclin D1 expression at the front of invasion than those with p53 wild-type pattern.

**Conclusion:**

The present study shows no differences in the expression of EMT markers between node positive and node negative vulvar cancers. The evaluation of immunostaining within the micro-anatomical context indicates that an EMT-phenotype is restricted to the tumor cells at the front of invasion. Paired analyses of vulvar carcinomas and their lymph node deposits suggest mesenchymal-epithelial transition (MET) in the metastatic deposits. Immunohistochemical staining results may suggest that EMT is more prevalent in vulvar cancer with aberrant p53 staining.

**Supplementary Information:**

The online version contains supplementary material available at 10.1007/s00432-021-03715-2.

## Introduction

Vulvar cancer is a rare gynaecologic malignancy accounting for 2–5% of all gynaecologic cancers (Rogers and Cuello 2018; Te Grootenhuis et al. 2018). Although it mostly affects elderly women, the incidence in younger patients is rising (Te Grootenhuis et al. 2018).

Vulvar squamous cell carcinoma (VSCC) is the most common histopathological subtype of vulvar cancer (Dasgupta et al. 2020; Rogers and Cuello 2018; Singh and Gilks 2020). There are two distinct molecular pathways that lead to VSCC, associated with either (1) VIN of the usual type (u-VIN) which is associated with high-risk HPV infections, mostly resulting in non-keratinizing VSCC and (2) VIN of the differentiated type (d-VIN), associated with p53 alterations often developing in a background of lichen sclerosus, mostly resulting in keratinizing VSCC (Dasgupta et al. 2020; Singh and Gilks 2020).

Regardless of the pathogenetic pathway, one of the most relevant prognostic factors for local recurrence of VSCC is the presence of inguinal lymph node metastases (Rogers and Cuello 2018; Te Grootenhuis et al. 2018). Risk factors for lymph node involvement of VSCC are the depth of invasion, tumor size and the ontogenetic tumor stage (Höckel et al. 2018; Julia and Hoang 2021) as well as morphologic features, such as infiltrative growth with dissociatively growing tumors and the presence of strong peritumoral desmoplastic change (Holthoff et al. 2016; Horn et al. 2012). Dissociative tumor growth and strong peritumoral stromal remodelling have been shown to be associated with epithelial-to-mesenchymal transition (EMT) in VSCC (Holthoff et al. 2016; Rodrigues et al. 2013). Furthermore, there is mounting evidence that EMT plays an essential role in lymphatic spread (Campo et al. 2015).

The present study was designed to evaluate the immuno-histochemical expression of EMT markers in correlation to the presence of inguinal lymph node involvement in VSCC.

## Materials and methods

Patients diagnosed with macro-invasive squamous carcinomas of the vulva were identified from our medical records. All patients had no previous vulvar surgery, had not received neoadjuvant therapy and were included in the prospective surgical trial for vulvar field resection based on the ontogenetic anatomy (German Clinical Trials Register, number DRKS00013358; (Höckel et al. 2018)). Patients were separated into those without and those with histopathologically proven inguinal lymph node involvement. Representative tumor tissue, using full slides, was evaluated immunohistochemically for p53, p16, MIB-1 and EMT markers (vimentin, cyclin D1, e-cadherin). Immuno-histochemical antibody information is provided in Table 1.

**Table 1:** Immuno-histochemical antibody information

Antibody	Clone	Vendor	Dilution and pretreatment	Detection system
p53	Do-7	Dako	1:100 CC1 36’/32’	DAB
p16	E6H4	Cintec Histology	ready to use CC1 36’/32’	DAB
cyclin D1	Sp4	Zytomed	1:50 CC1 64 ‘/32‘	DAB
vimentin	Vim3B4	Dako	1:200 CC1 36 ‘/32‘	Ultraview
e-cadherin	36b5	Novocastra	1:20 CC1 76 ‘/32‘	Ultraview
MIB-1	MIB-1	DAKO	1:100 CC1 64 ‘/32‘	DAB

p16 was scored as positive if there was diffuse block-like cytoplasmic and nuclear staining and as negative for any lesser staining (such as patchy or absent staining) in accordance with the LAST (Lower Anogenital Squamous Terminology) project recommendations (Darragh et al. 2013) and recent studies (Tessier-Cloutier et al. 2020).

p53 was scored as wild type representing heterogeneous nuclear staining of variable intensity of scattered nuclear-positive tumor cells in the basal and parabasal layers and mid-epithelial with heterogeneously positively stained nuclei with notable basal negativity of the invasive tumor cell nests. Mutant staining patterns were defined as strong nuclear staining in at least 80% of the basal cells with or without significant parabasal extension, complete negative staining of the tumor cells with the presence of a positive staining in adjacent inflammatory and stromal cells (serving as positive internal control) as described recently for VSCC (Tessier-Cloutier et al. 2020).

**Fig. 1:**
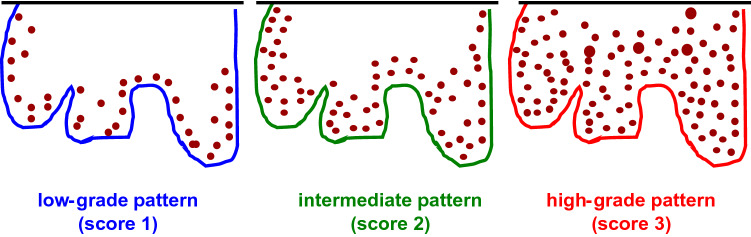
Schematic illustration of Ki-67 scoring system: Score 1 = low-grad**e** pattern: positive staining of the outer cell layers of the invasive tumor cells nests with very limited suprabasal extension. Score 2 = intermediate pattern: positive staining up to the mid of the infiltrating tumor cell nests. Score 3 = high-grade pattern: with diffuse or near-diffuse staining of the tumor cell nests

Nuclear staining of Ki-67 was scored from 1–3 (Fig. 1) on the basis of a comparison with normal vulvar squamous epithelium (Podoll et al. 2017) and existing evidence on penile squamous cell carcinoma (Protzel et al. 2007; Stankiewicz et al. 2012): 1 = basal expression or low-grade pattern with nuclear Ki-67 staining of the basal/ outer cell layers of the invasive tumor cells nests with very limited suprabasal extension; 2 = increased expression or intermediate pattern with staining up to the middle of the infiltrating tumor cell nests; 3 = increased expression or high-grade pattern with diffuse staining of the tumor cell nests. All nuclear staining was recognised as positive, regardless of the staining intensity.

For EMT markers, an immuno-reactive score (IRS) was calculated by summarizing the percentage of positive cells and their staining intensity. Staining intensity (SI) was scored as weak, moderate or strong. The percentage of positive tumor cells at the front of invasion was scored as 0 = 0% positive tumor cells, 1 = 1% positive cells, 2 = 2– < 10% positive cells, 3 = 10– < 33% positive cells, 4 = 33– < 66% positive cells, 5 = 66–100% positive cells. The median values of IRS were evaluated in correlation to the presence of inguinal lymph node involvement. Additionally, the zonal staining pattern was evaluated, comparing the front of invasion with the tumor center (Stewart and McCluggage 2013).

The pattern of invasion and the grade of peritumoral desmoplastic reaction (DSR) and inflammatory response (PER) were evaluated in correlation to the lymph node status as previously described (Horn et al. 2012).

The study cohort was described by standard descriptive statistics: median for continuous, number for categorical variables. Relationships between categorical variables were analysed by crosstabs, chi-squared tests and Fisher's exact tests as appropriate. The location was compared by nonparametric tests due to the low sample size and asymmetrical distribution of the scores, that is, Mann–Whitney *U* test for two and Kruskal–Wallis test for more than two groups.

Median differences including 95% confidence intervals were calculated as effect measures following Hodges and Lehman. The significance level was determined at 5% for two-tailed tests. All analyses were performed by IBM SPSS Statistics, version 26.

An Institutional Review Board approval was obtained prior to the initiation of the study.

## Results

### Clinicopathological data

Thirty-two cases were included in the analysis. Their clinico-pathological characteristics are summarised in Table 2.

**Table 2:** Patient characteristics

	Inguinal lymph node status			
	All cases (N = 32)	pN0 (*N* = 16)	pN1 (*N* = 16)	
Median age (years)	70.7 (39.8–87.5)	68.6 (39.8–77.7)	74.3 (40.5–87.5)	*p* = 0.017
Tumor stage				
pT1b	16 (50.0%)	8 (50.0%)	8 (50.0%)	
pT2	10 (31.2%)	4 (25.0%)	6 (37.5%)	
pT3	6 (18.8%)	4 (25.0%)	2 (12.5%)	*p* = 0.59
Tumor grade				
G1	2 (6.3%)	2 (12.5%)	0	
G2	20 (62.5%)	9 (56.3%)	11 (68.8%)	
G3	10 (31.2%)	5 (31.2%)	5 (31.2%)	p = 0.61
Peritumoral desmoplastic change				
None	4 (12.5%)	2 (12.5%)	2 (12.5%)	
Weak	10 (31.3%)	5 (31.3%)	5 (31.3%)	
Moderate	3 (9.3%)	1 (6.2%)	2 (12.5%)	
Strong	15 (46.9%)	8 (50.0%)	7 (43.7%)	p = 1.0
Peritumoral inflammatory response				
None	8 (25.0%)	4 (25.0%)	4 (25.9%)	
Weak	6 (18.8%)	1 (6.3%)	5 (31.3%)	
Moderate	10 (31.2%)	7 (43.8%)	3 (18.7%)	
Strong	8 (25.0%)	4 (25.0%)	4 (25.0%)	*p* = 0.26
Pattern of invasion				
Spray-like	19 (59.4%)	10 (62.5%)	9 (56.3%)	
Finger-like	13 (40.6%)	6 (37.5%)	7 (43.8%)	*p* = 1.0
p53-staining^a^				
Aberrant	20 (64.5%)^b^	11 (68.7%)	9 (60.0%)	
Wild type	11 (35.5%)	5 (31.3%)	6 (40%)	*p* = 0.61
p16-staining^a^				
Block	12 (38.7%)^b^	5 (31.2%)	7 (46.7%)	
Non-block	19 (61.3%)	11 (68.7%)	8 (53.3%)	*p* = 0.38
Ki-67-staining^a^				
Score 1	16 (51.6%)	10 (66.7%)	6 (40.0%)	
Score 2	7 (22.6%)	3 (20.0%)	4 (26.7%)	
Score 3	8 (25.8%)	3 (13.3%)	5 (33.3%)	*p* = 0.52

^a^In one case no tumor tissue was left within the only available tumor containing block for immunostaining

^b^One single case represented aberrant p53 expression as well as block-p16-positivity within the tumor (Fig. 4c,d)

Patients with inguinal lymph node involvement were in median six years older compared to patients without metastatic spread (68.6 vs. 74.3 years; Table 2).

**Fig. 2:**
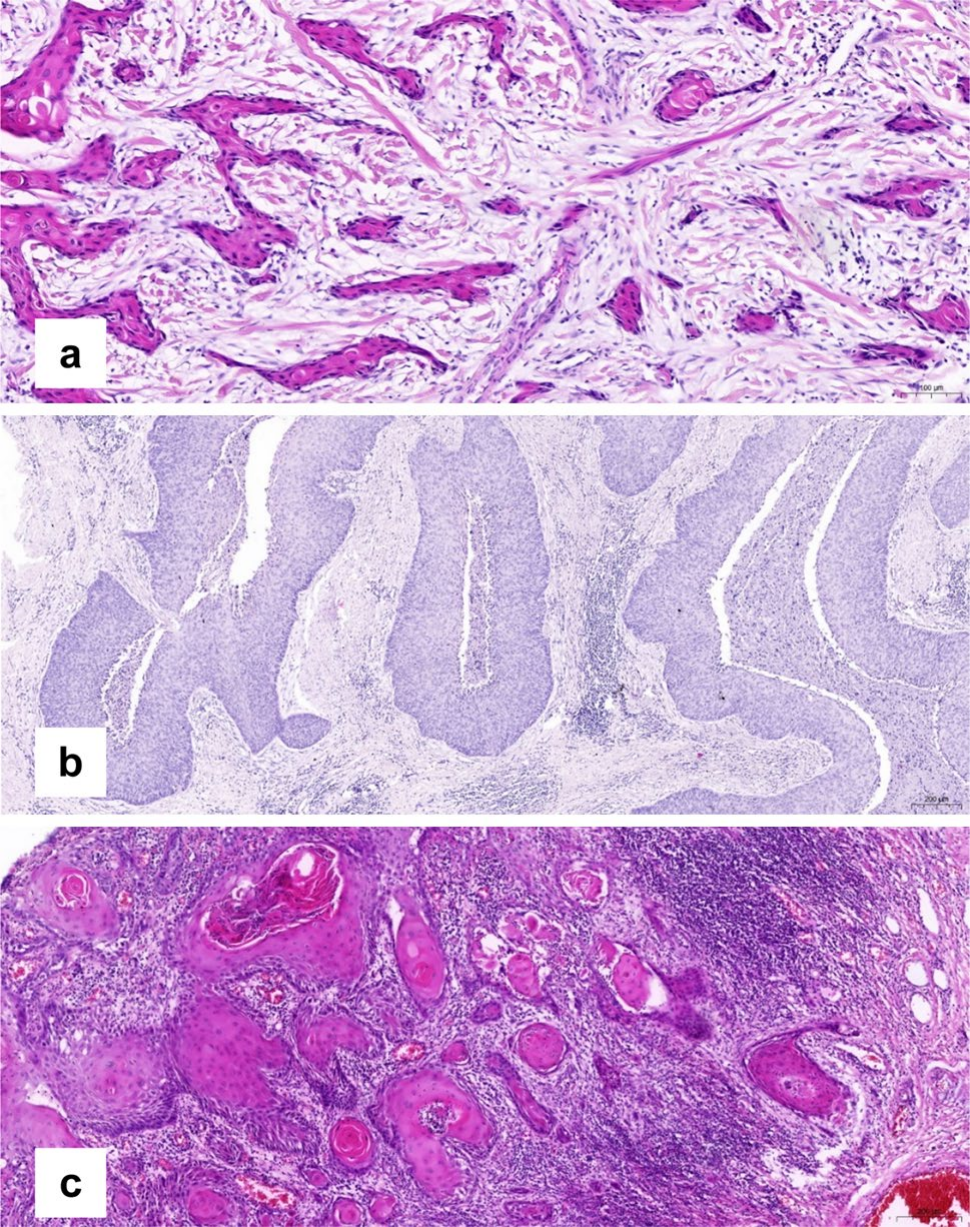
Pattern of invasion and peritumoral stromal remodelling of VSCC. **a** Spray-like pattern of invasion and strong peritumoral desmoplastic reaction but no peritumoral inflammatory response. **b** Finger-like pattern of invasion without peritumoral desmoplastic reaction, very weak peritumoral inflammatory response. **c** Keratinizing VSCC with strong peritumoral inflammatory response

About one half of the tumors showed strong peritumoral desmoplastic change (DES; Fig. 2a). There were no differences between the grade of DES when node positive and negative cases were compared (*p* = 1.0).

56.3% of the node positive tumors showed no or an only weak peritumoral inflammatory response (PER; Fig. 2b), whereas 68.8% of the carcinomas without inguinal lymph node involvement (pN0) represented with a moderate or strong PER (Fig. 2c) without reaching statistical significance (*p* = 0.26). The different patterns of invasion were not associated with the lymphatic spread (Table 2).

### Proliferate activity and p53- and p16-immunostaining

**Fig. 3:**
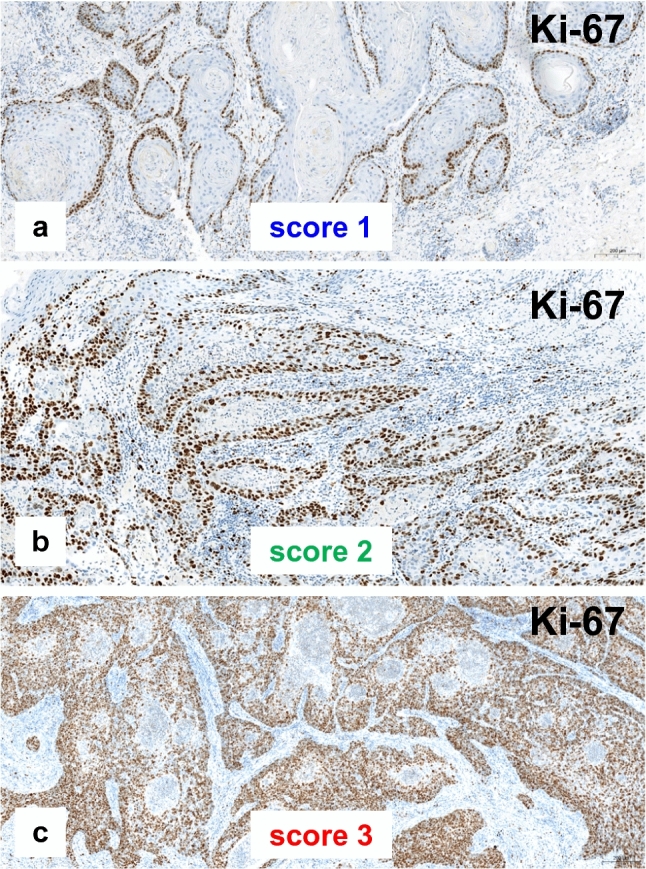
Different Ki-67 scores. **a**, **b** Keratinizing VSCC with low proliferative activity. **c** Non-keratinizing VSCC with very high proliferative activity

More than 60% of the VSCC without inguinal lymph node involvement showed a low proliferative activity (Ki-67 score 1), whereas one-third of the tumors with positive inguinal nodes were highly proliferative, with a Ki-67 score of 3 (Table 2, Fig. 3).

**Fig. 4:**
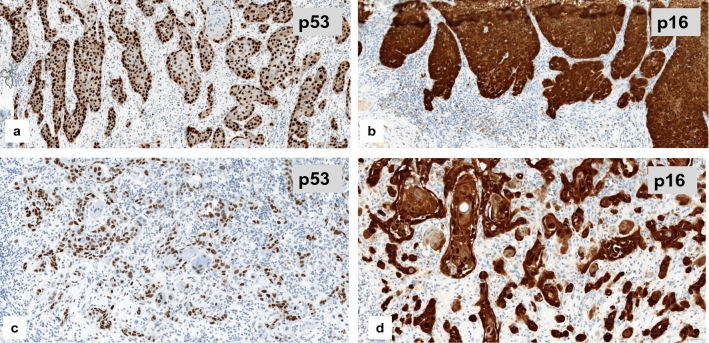
p53- and p16-immunostaining as surrogate marker for the different pathogenetic pathways of VSCC. **a** Diffuse nuclear overexpression of p53 (i.e. aberrant expression), suggestive of a p53 mutation. **b** Diffuse staining for p16 (block staining), suggestive for HPV high-risk-associated tumor (**c**) and (**d**) so-called double classifier within a keratinizing VSCC, representing both an aberrant p53 expression and block staining for p16 within the same tumor

About two-thirds of tested VSCC represented aberrant p53 staining which is suggestive of a p53 mutation (Tessier-Cloutier et al. 2020; Table 3, Fig. 4a). One-third showed p16 block staining which has been reported to be associated with high-risk HPV infections (Tessier-Cloutier et al. 2020; Table 3, Fig. 4b). One single case showed both, aberrant p53 expression accompanied by block-p16-positivity (Fig. 4c), representing a so-called double classifier (Tessier-Cloutier et al. 2020). That special case was included in further analysis of the EMT-marker expression as p16-positive one.

**Table 3:** Results of p53- and p16-immunostaining in correlation to proliferative activity (*N* = 31^a^)

	p16^non−block^/p53^aberrant^	p16^block^/p53 ^wt^	
Number of cases	20 (64.5%)	11 (35.5%)	
Proliferative activity (Ki-67)			
Score 1	14 (70.0%)	2 (18.2%)	
Score 2	5 (25.0%)	2 (18.2%)	
Score 3	1 (5.0%)	7 (63.6%)	*p* < 0.005

^a^All cases of the study could be stained immunohistochemically for vimentin and cyclin D1. In one case no tumor tissue was left within the only available tumor containing block for Ki-67 staining

**Fig. 5:**
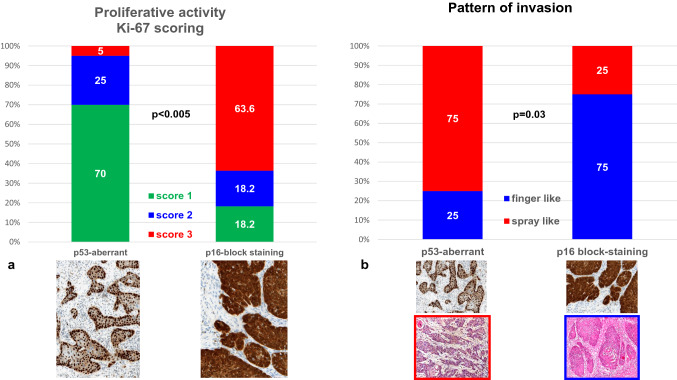
Proliferative activity (Ki-67 scoring) and different patterns of invasion in correlation to immuno-histochemical p16- and p53-expression as surrogate marker for different pathogenetic pathways in VSCC (see text)

Although a limited number of cases were included in this study, those with an immuno-histochemical signature of p16^block^/p53^wt^, indicating an HPV association, represented a significantly higher number of highly proliferative tumors characterized by a Ki-67 score of 3 (Fig. 5a). In contrast, the p16^non−block^/p53^aberrant^ tumors which may be associated with a p53 mutation, showed a low proliferative activity based on a Ki-67 score of 1 (Fig. 5a; Table 3). There were significantly more spray-like patterns of invasion in the p53-aberrant compared to the p16-block-positive tumors (Fig. 5b).

### Immunostaining of EMT markers

**Fig. 6:**
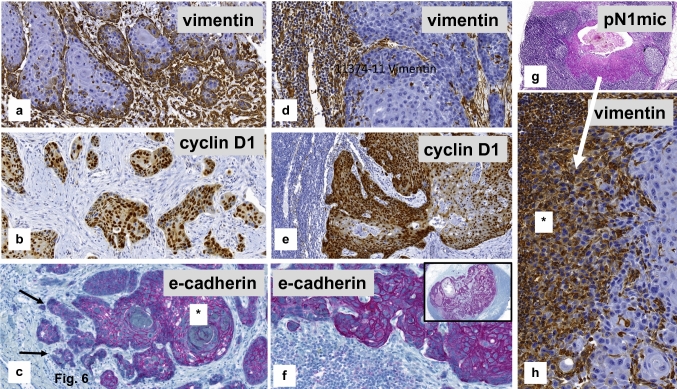
Immuno-expression of EMT markers within vulvar primary and inguinal lymph node metastases. **a**–**c** Primary VSCC with cytoplasmatic expression of vimentin within infiltrative tumor cell nests (**a**), nuclear staining for cyclin D1 (**b**), strong (*) and reduced/negative (arrow) membranous staining for e-cadherin (**c**), indicating *epithelial–mesenchymal transition (EMT).* (**d**–**f**) Tumor deposits within lymph node metastases representing negative staining for vimentin, nuclear positivity for cyclin D1 and diffuse staining for e-cadherin, indicating *mesenchymal-epithelial transition (MET)* (**g**, **h**) Micro-metastatic deposit within an inguinal lymph node (**a**) representing strong cytoplasmatic staining for vimentin of the majority of tumor cells (*)

No differences within the median values of the immuno-reactive scores (IRS) could be proven when comparing node negative and positive cases for vimentin [pN0: 2.5 (95% CI: 0.0–6.5) versus pN1: 3.5 (95% CI: 0.0–5.5); *p* = 0.93] and cyclin D1 [pN0: 5.5 (95% CI: 3.0–7.5) versus pN1: 5.5 (95% CI: 4.0–7.5); *p* = 1.0] or for e-cadherin (*p* = 1.0). Characteristic staining results of the different EMT markers within the VSCC are illustrated in Figs. 6a-c.

Although not reaching statistical significance, VSCC with aberrant p53 staining showed a higher vimentin expression within the tumor cells (*p* = 0.087). In contrast, p53-aberrant tumors showed an increased cyclin D1 expression compared to p16-positive tumors (*p* = 0.027; suppl. Table 1), while no difference was noted for e-cadherin (*p* = 1.0).

Evaluating the *EMT markers within the lymph node metastases*, positive staining was seen in all nodal tumor deposits for e-cadherin with an average of 74.9% positive tumor cells. While one case was completely negative for cyclin D1, positive staining was observed an average of 48.7% of positive stained tumor cells in all other cases (Fig. 6d–f). Only three metastatic deposits within the inguinal lymph nodes showed positive staining for vimentin. One of these cases presented with a very small metastasis of 0.25 mm in largest dimension (Fig. 6g, h). That staining pattern may suggest mesenchymal–epithelial transition (MET) within the metastatic tumor cells in the lymph node deposits.

**Fig. 7:**
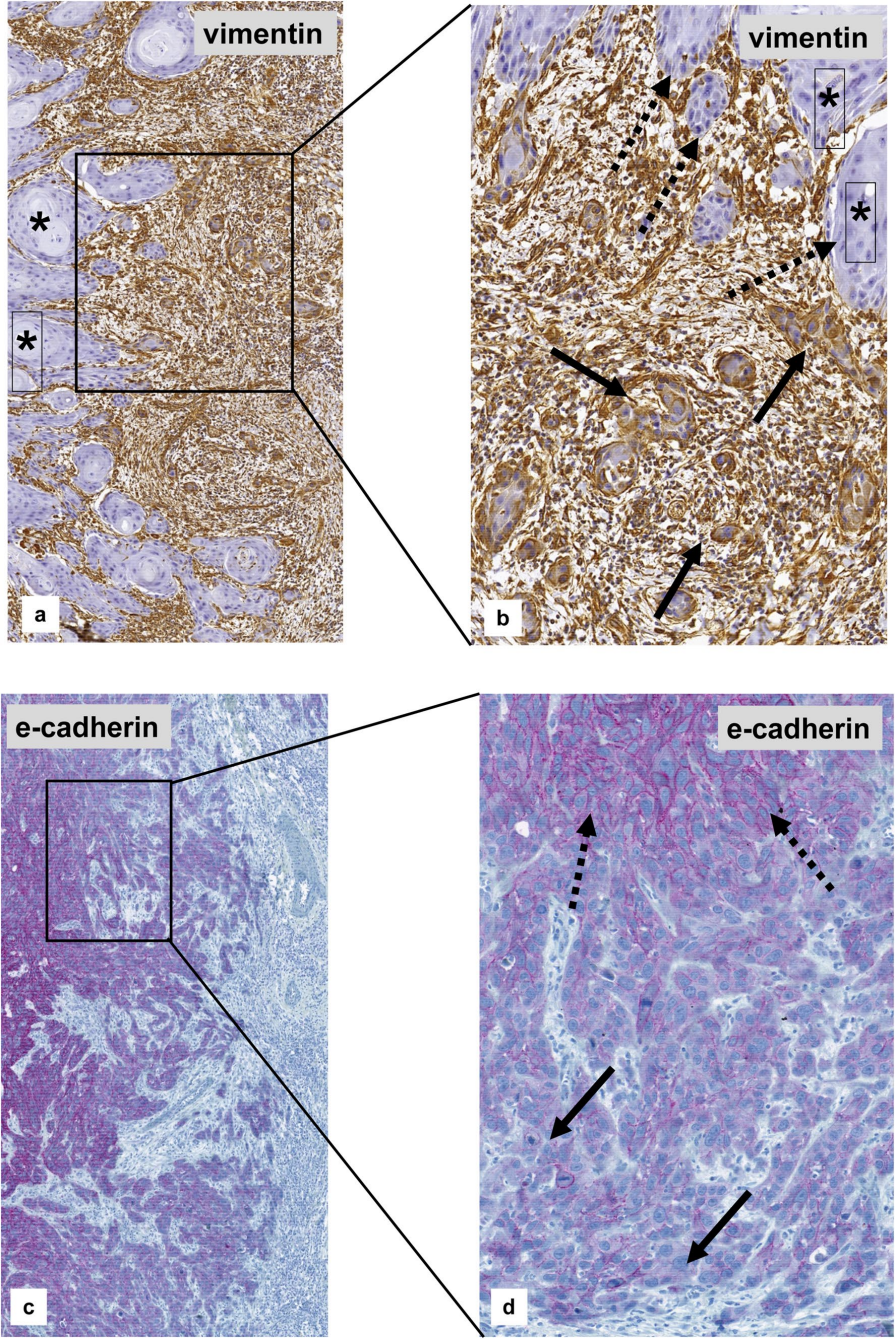
Micro-anatomical EMT-immunostaining of a topographic subpopulation of tumor cells within the primary vulval VSCC. **a**, **b** Strong cytoplasmatic staining for vimentin at the front of invasion (arrow), whereas tumor cells of the tumor center are negative (*). **c**, **d** Strong immunostaining for e-cadherin within the center of the tumor (dotted arrow), but negative or strongly reduced membranous staining at the front of invasion (arrow)

Evaluating the zonal staining within the tumor, positive staining for vimentin was restricted to the front of invasion in all cases, not the center of the tumor (Fig. 7a, b). The majority of cases (27/32; 84.4%) showed positive staining for cyclin D1 at the front of invasion whereas the center of the tumor was negative. The opposite was observed for e-cadherin. More than two-thirds of the cases (22/32; 68.7%) represented negative or reduced tumor cell staining at the front of invasion (Fig. 7c, d) compared to the center of the tumor.

No correlation between the tested EMT markers and the grade of peritumoral desmoplastic change, inflammatory response or the different patterns of invasion could be proven (suppl. Table 2–4). The spray-like pattern of invasion was significantly more common in p16^non−block^/p53^aberrant^ tumors compared to p16^block^/p53^wt^ tumors (15/20 (75%) vs. 3/12 (25%); *p* = 0.009; suppl. Table 1, Fig. 5b).

## Discussion

Lymph node involvement and recurrent disease play a critical role in the prognosis of VSCC (Rogers and Cuello 2018; Te Grootenhuis et al. 2018). Depending on the tumor stage, up to 50% of all VSCC patients will be present with lymph node involvement at the time of diagnosis (Höckel et al. 2018). Among those node positive patients, 30–50% will relapse (Melo Maia et al. 2013).

Epithelial–mesenchymal transition (EMT) has been studied in several malignancies (Bhangu et al. 2012; Toll et al. 2013), including gynecologic cancers (Stewart and McCluggage 2013). However, only few studies are available for vulvar cancer (Holthoff et al. 2016; Rodrigues et al. 2013; Zannoni et al. 2011). EMT represents a molecular shift within the tumor cells, leading to the adoption of a mesenchymal phenotype associated with loss of polarity, loss of cell adhesion and enhanced cellular motility (Toll et al. 2013), with a consecutively increased risk of metastatic spread and poor prognosis (Bhangu et al. 2012; Stewart and McCluggage 2013; Toll et al. 2013). While many immuno-histochemical markers (IHC) have been evaluated to identify cells undergoing EMT, some of the most validated markers include vimentin, e-cadherin and cyclin D1 (Holthoff et al. 2016; Stewart and McCluggage 2013; Zannoni et al. 2011).

It has been shown that EMT markers are highly upregulated in VSCC tumors representing a spray-like pattern of invasion (Holthoff et al. 2016), mostly associated with a strong peritumoral desmoplastic reaction and low peritumoral inflammatory response. As reported for squamous cell carcinomas of the uterine cervix (Horn et al. 2012), a spray-like pattern of invasion is also associated with a higher risk of lymphatic spread and poor prognostic outcome in VSCC (Holthoff et al. 2016; Jeffus et al. 2015). These results could not be confirmed examining our cases (Tab. 1), may be done by the limited number of cases examined. There was no correlation of the expression of the tested EMT markers (vimentin, cyclin D-1, e-cadherin) when the different patterns of invasion were compared. The missing correlation may be biased by the limited number of cases tested.

It has been shown that cells with an EMT phenotype are able to escape the immunological surveillance by host immune cells (Kudo-Saito et al. 2009). Holthoff et al. (2016) reported that VSCC with a strong peritumoral *desmoplastic* reaction displayed a 3.3-fold increase in the number of cases with positive staining for vimentin compared to patients with a strong peritumoral *inflammatory* reaction (*p* = 0.0031). In contrast, Rodrigues et al. (2013) described no correlation between the expression of e-cadherin, vimentin and other EMT-related markers and the inflammatory response in VSCC. In the present study, there was no correlation between the grade of peritumoral inflammatory response or the desmoplastic reaction and the expression of the tested EMT markers within the tumor cells (suppl. Table 2–4). In the study of Holthoff et al. (2016), there was no correlation between the loss of e-cadherin and peritumoral stromal response in VSCC. Regarding the contradictory results in a potential correlation between the peritumoral inflammatory response and the expression of EMT markers in VSCC, it may be hypothesized, that the presence of strong inflammatory response at the front of invasion may suppress the process of EMT in squamous cell carcinomas (Holthoff et al. 2016). Results supporting this idea are pending.

Examining HPV-negative and -positive cell lines from squamous cell carcinomas, it has been shown that HPV-/p16-positive cell lines represented a reduced EMT transition (Umbreit et al. 2014).

Rodrigues et al. (2013) described no correlation between e-cadherin and vimentin and a potential HPV infection in VSCC. However, a low β-catenin and high Slug expression were significantly associated with HPV-negative (p53-aberrant) tumors and a poor prognostic outcome. Although not reaching statistical significance, the median IRS value of vimentin was higher in VSCC with aberrant p53 staining when compared to wild type staining (4.5 vs. 0; *p* = 0.087; suppl. Table 1). For cyclin D1, a significantly increased staining was seen in p53-aberrant VSCC (*p* = 0.03). These results may indicate that different EMT markers may be expressed differently in VSCC depending on its pathogenetic pathways (HPV high-risk infection versus p53 mutation). In the present study, p16^non−block^/p53^aberrant^ tumors, were associated with a spray-like pattern of invasion and low proliferative activity (Ki-67 labeling score 1; Fig. 5a), likely caused by the p53 mutation. In contrast, in p16^block^/p53^wt^ VSCC, indicating a high-risk HPV infection, finger-like pattern of invasion and high proliferative activity (score 3 Ki-67 labeling index) were significantly more common (Fig. 5b).

In squamous cell cancer of the head and neck as well as in those of the female genital tract, it has been suggested that upregulated EMT may be associated with a higher tumor stage and the presence of metastatic spread (Stewart and McCluggage 2013; Wan et al. 2020). Holthoff et al. (2016) were unable to demonstrate any correlation between the immuno-histochemical expression of the EMT markers β-catenin, vimentin and e-cadherin and nodal involvement in VSCC. Low e-cadherin expression was associated with deeper invasion, higher tumor stage and ≥ 2 positive lymph nodes in VSCC (Rodrigues et al. 2013). In the same study, there was a significant association between positive vimentin staining and the depth of invasion, but as mentioned above, not with lymph node involvement. In the present study, there were no differences in the immuno-reactive scores of vimentin and cyclin D1 or the staining of e-cadherin between node negative and node positive cases. In the authors’ opinion, additional studies are required examining a larger number of cases to compare node negative and positive VSCC. Furthermore, the cases need to be separated based on the different pathogenetic pathways of VSCC (high-risk HPV- and p53-driven tumors).

In the present study, different microanatomical locations, i.e. topographic subpopulations of tumor cells were evaluated regarding the immuno-histochemical expression of EMT markers comparing the central tumor to its front of invasion. The vast majority of cases demonstrated pronounced cyclin D1 staining at the front of invasion while vimentin staining was seen exclusively in tumor cells at the invasion front (Fig. 7a, b). Contrary, more than two-thirds of the cases represented reduced or negative staining for e-cadherin at the front of invasion (Fig. 7c, d). Holthoff et al. (2016) reported that immuno-histochemical expression of vimentin was exclusively seen at the front of invasion in two-thirds of VSCC, and 77% displayed a loss of e-cadherin. In head and neck squamous cell carcinomas, Dal Vecchio et al. (2011) reported a significant correlation between the expression of vimentin and lymph node involvement. Rodrigues et al. (2013) found an increased vimentin expression at the front of invasion for vulvar carcinomas. In the same study, a significant loss of nuclear β-catenin staining (*p* = 0.013) was seen in tumor cells at the front of invasion, especially in HPV-negative VSCC. The infiltrative tumor cells in CIN 3-like squamous cell carcinomas of the uterine cervix showed a nuclear expression of cyclin D1 and loss of e-cadherin (Stewart and Crook 2017) whereas the central parts of the tumor demonstrated a reverse staining pattern. Both nuclear expression of cyclin D1 and loss of e-cadherin were also pictured at the front of invasion within the review by Stewart and MaCluggage (2013). Zannoni et al. (2011) reported a down-regulation of e-cadherin expression in VSCC compared to normal squamous epithelium at the front of invasion. Loss of e-cadherin leads to reduced cell adhesion which may result in an increased metastatic potential of tumor cells (Bakir et al. 2020; Jolly et al. 2017). The different zonal staining patterns suggest that the tumor cells at the front of invasion acquire an invasive and migratory behavior to support the infiltrative tumor growth and support the ability for metastatic spread. Different microanatomical staining patterns for EMT-associated markers have been reported for several gynecologic malignancies (Stewart and McCluggage 2013) including cervical squamous cell carcinomas (Koay et al. 2012; Lee and Shen 2012), suggesting that the EMT phenotype of cancer cells is a prerequisite for infiltrative tumor growth at the front of invasion as well as metastatic spread (Bakir et al. 2020; Jolly et al. 2017).

Meanwhile, EMT is believed to be a reversible process, affected by tumor microenvironment, and is followed by an inverse process, resulting in a mesenchymal–epithelial transition (MET; Bakir et al. 2020; Jolly et al. 2017; Stewart and McCluggage 2013). One hypothesis is that MET may be necessary for colonisation and tumor growth at the metastatic site (Guarino et al. 2007; Stewart and McCluggage 2013) as well as the realisation of metastatic deposits from circulating tumor cells (Bakir et al. 2020; Jolly et al. 2017). These ideas may be supported by the present study, showing an immuno-histochemical expression of e-cadherin in the tumor cells with on average 74.9% positively stained cells in inguinal lymph metastases of VSCC, accompanied by tumor cells that are negative for vimentin in 12/16 cases within the lymph node deposits. Additionally, one of the cases with a small tumor cell deposit within the lymph node was positive for vimentin (Fig. 6h), whereas the larger metastases were completely negative. However, the number of cases included in the present study was too small to draw definitive conclusions.

The present study showed no differences in the expression of EMT markers when node positive and negative VSCC were compared. However, different staining patterns were noted within different cellular subpopulations in their micro-anatomical context indicating an EMT-phenotype in the tumor cells at the front of invasion. Paired analyses of tumor cells within the vulvar tumor and its metastatic deposits within the inguinal nodes may indicate a mesenchymal–epithelial transition (MET). Although a limited number of cases was examined, our results and those obtained from the literature suggest that EMT may be more frequent in tumors associated with a p53 mutation compared to those caused by a high-risk HPV infection. The pronounced EMT features in VSCC representing p53 alterations may be responsible for the more aggressive clinical behaviour of that tumors, regardless of lower proliferative activity.

Additional studies are required examining a larger cohort of cases with separation VSCC according to its different pathogenetic pathways. A better understanding of the etiology for lymphatic spread and metastases in HPV- and non-HPV-associated vulvar squamous cell carcinoma may eventually help identify patients at high risk for lymphatic or metastatic spread.

## Supplementary Information

Below is the link to the electronic supplementary material.

Supplementary file1 (DOCX 18 kb)
