# Supplementary material for: Epithelial–mesenchymal transition (EMT) in vulvar cancer with and without inguinal lymph node involvement
Source: J Cancer Res Clin Oncol. 2021 Sep 8;148(5):1183–93. doi: 10.1007/s00432-021-03715-2 (PMC9015993; doi:10.1007/s00432-021-03715-2)
Supplement: Supplementary file 1 — Supplementary file1 (DOCX 18 kb) [file 432_2021_3715_MOESM1_ESM.docx]

Supplementary Table 1: Results of p53- and p16-immunostaining in correlation to EMT-marker expression

_________________________________________________________________________________

**p16^non-block^/p53^aberrant^ p16^block^/p53 ^wt^**

_________________________________________________________________________________

**vimentin** (median value of immunoscore)

4.5 0 p=0.087

**cyclin D1** (median value of immunoscore)

7 5 p=0.03

**e-cadherin** (zonal staining results)

homogenous^1^ 14 (70.0%) 8 (72.2%)

invasion front

negative 6 (30.0%) 3 (27.3%) p=1.0

**pattern of invasion**

finger-like 5 (25.0%) 9 (81.8%)

spray-like 15 (75.0%) 2 (18.2%) p<0.03

^1^homogenous staining results indicating positive stained tumor cells within the center of the tumor AND tumor cells at the front of invasion

Supplementary Table 2: Correlation between peritumoral inflammatory response and immunostaining for EMT markers

**Grade of peritumoral *inflammatory* response**

**none weak moderate strong**

**vimentin** (median value of immunoscore)

1.5 0.0 4.5 5.0 p=0.40

**cyclin D1** (median value of immunoscore)

4.0 6.5 6.5 4.5 p=0.29

**e-cadherin** (zonal staining results)

homogenous^1^ 6 (85.7%) 5 (83.3%) 7 (70.0%) 4 (50.0%)

invasion front p=0.46

negative 1 (14.3%) 1 (16.7%) 3 (30.0%) 4 (50.0%)

^1^homogenous staining results indicating positive stained tumor cells within the center of the tumor AND tumor cells at the front of invasion

Supplementary Table 3: Correlation between peritumoral desmoplastic reaction and immunostaining for EMT markers

**Grade of peritumoral *desmoplastic* reaction**

**none weak moderate strong**

**vimentin** (median value of immunoscore)

2.0 2.5 5.00 3.0 p=0.50

**cyclin D1** (median value of immunoscore)

3.0 4.5 7.0 7.0 p=0.05.

**e-cadherin** (zonal staining results)

homogenous^1^ 3 (75.0%) 8 (80.0%) 2 (66.7%) 9 (64.3%)

invasion front p=0.50

negative 1 (25.0%) 2 (20.0%) 1 (33.3%) 5 (35.7%)

^1^ homogenous staining results indicating positive stained tumor cells within the center of the tumor AND tumor cells at the front of invasion

Supplementary Table 4: Correlation between different patterns of invasion and immunostaining for EMT markers

**Pattern of invasion**

**finger-like spray-like**

**_________________________________________________________________________________**

**vimentin** (median value of immunoscore)

4.0 3.0 p=1.0

**cyclin D1** (median value of immunoscore)

4.0 7.0 p=0.027

**e-cadherin** (zonal staining results)

homogenous^1^ 10 (76.9%) 12 (66.7%)

invasion front p=0.70

negative 3 (23.1%) 6 (33.3%)

^1^ homogenous staining results indicating positive stained tumor cells within the center of the tumor AND tumor cells at the front of invasion
